# Iowa Gambling Task performance in individuals with schizophrenia: the role of general versus specific cognitive abilities

**DOI:** 10.3389/fpsyt.2024.1454276

**Published:** 2024-12-10

**Authors:** Stian Orm, Merete Glenne Øie, Ingvild Haugen

**Affiliations:** ^1^ Department of Research, Innlandet Hospital Trust, Brumunddal, Norway; ^2^ Department of Psychology, Inland Norway University of Applied Sciences, Lillehammer, Norway; ^3^ Department of Psychology, University of Oslo, Oslo, Norway

**Keywords:** Iowa Gambling Task, decision making, executive function, schizophrenia, psychosis

## Abstract

**Objective:**

We aimed to explore how specific cognitive processes, such as attention and executive functions, account for variance in decision-making measured by Iowa Gambling Task (IGT) performance among individuals with schizophrenia spectrum disorders.

**Methods:**

Adults (*N* = 65, *M*_age_ = 25.4) with schizophrenia spectrum disorders participating in a clinical trial (registered at clinicaltrials.gov NCT03048695) completed the IGT, neuropsychological tests of attention, response inhibition, mental flexibility, working memory, and planning, as well as subtests from the Wechsler tests of intelligence to estimate IQ. Associations between performance on specific tasks, a composite score of executive function and attention, and IGT performance measured in two ways, one using the total net score, decks (C+D) – (A+B) and the other as preference for decks with more frequent gains than losses, decks (B+D) – (A+C), were analyzed with correlational and hierarchical regression analysis controlling for estimated IQ and psychotic symptoms, measured by the Positive and Negative Syndrome Scale.

**Results:**

In the regression analyses, the strongest predictor of IGT performance measured as the total net score was estimated IQ (*b* = 1.43, *p* <.001). Neither specific cognitive tasks nor the composite score of executive functioning significantly contributed to explaining variance in IGT total net score beyond IQ and symptoms of psychosis. However, IQ and symptoms of psychosis did not predict tendency towards selecting decks with different gain-to-loss frequency, whereas poorer composite executive functioning predicted a pattern of selecting decks A and C with more frequent losses, (*b* = 8.30, *p* <.05).

**Discussion:**

The results suggest that both IQ and executive functions contribute to IGT performance, but in distinct ways. Whereas lower IQ may contribute to overall more disadvantageous decision-making, poorer executive functioning may contribute to a more risk-aversive decision-making style. A clinical implication may be that individuals with schizophrenia and lower IQ or poorer executive functioning will have a higher need for support and interventions targeting decision-making.

## 1 Introduction

Schizophrenia is commonly recognized as one of the most severe mental disorders, characterized by hallucinations and delusions (i.e., positive symptoms) as well as diminished emotional expressions, avolition, and social withdrawal (i.e., negative symptoms) (1, 2). The lifetime prevalence of schizophrenia in the population is between 0.7% and 0.9% (3, 4). Despite the low prevalence, schizophrenia represents a heavy burden in terms of healthcare costs and years lived with disability (5, 6). Thus, research aimed at better understanding the multidimensional nature of schizophrenia and factors associated with heterogeneity in functioning is important for clinical care.

Despite existing treatment for psychotic symptoms, a substantial portion of individuals with schizophrenia experience reduced real-world function (7, 8). As cognition has proved a significant predictor of function, it has become an important treatment target (9, 10). Individuals with schizophrenia commonly present with poorer cognitive performance across a range of different domains (11). Lower pre-onset IQ predicts a higher risk of schizophrenia onset (12, 13). However, despite this association between lower IQ and schizophrenia risk, there is considerable variation in cognitive performance among individuals with schizophrenia, and almost one-fourth (22%) perform averagely or above averagely on tests of IQ (14). Beyond this deficit in global cognitive abilities, individuals with schizophrenia also commonly display difficulties across a range of different specific cognitive tasks, including attention, inhibition, working memory, shifting, and planning (15–20). These cognitive processes are commonly referred to as executive functions, an umbrella term for cognitive processes involved in the control of cognition, emotion, and behaviors (17, 21). The cognitive difficulties with executive functions and IQ have been found to be unrelated to positive symptoms and only weakly or moderately related to negative symptoms (22–24). Thus, the cognitive difficulties represent an important clinical domain, independent of the core symptoms of schizophrenia. Furthermore, demonstrating the importance of executive functions for clinical outcomes, studies have found that difficulties with executive functions in individuals with schizophrenia predict more functional impairments and internalizing difficulties later in life (25, 26).

Another domain where individuals with schizophrenia display poorer performance than their healthy counterparts is decision-making (27, 28). Individuals with schizophrenia more often display risk-taking behaviors, including among others substance-use and criminal offences (29–31). These risk-taking behaviors may be tied to decision-making processes (32). The Iowa Gambling Task (IGT; 33) is one laboratory task that has been extensively used in the study of decision-making processes in clinical and non-clinical populations (27, 28, 34–36). The IGT is a simulation of a situation where participants can win and lose money by drawing cards without knowing up front which decks of cards are more beneficial. The implicit nature of the task separates it from explicit tasks where probabilities are made explicit from start. Decision-making under implicit contingencies is considered to include a “hot” aspect, referring to the affective response participants have to the choice options (33, 37). The somatic marker hypothesis suggests that reactivation of bodily responses (e.g. increase in heart rate or sweat) to previous losses help guide decision-making (38, 39). Explicit tasks are not assumed to cause the same affective responses, but are rather considered “cold” in the sense that participants can make rational decisions about risk and benefit based on the known probabilities of the task (37).

Two meta-analyses have demonstrated that individuals with schizophrenia exhibit poorer performance on the IGT, i.e. they chose the disadvantageous decks more often (27, 28). This finding indicate that individuals with schizophrenia have difficulties in deciphering the risk/reward contingencies of the task and may struggle to adjust their strategy based on feedback. Generally, IGT performance has inconsistently been found to be positively related with IQ and executive functions (34). However, in one study of healthy individuals, IQ could account for around 40% of the variance in IGT performance and attention and shifting could account for 37% and 17% of the variance in IGT performance, respectively (40). Similarly, one of the meta-analysis of IGT performance in individuals with schizophrenia found a significant positive correlation with IQ (*r* = .20) and working memory (*r* = .22), whereas evidence for an association with overall executive functioning was inconclusive (27). The other meta-analysis of IGT performance in individuals with schizophrenia, however, found that whereas higher IQ was associated with placing lesser weight on immediate gain and increased weighting of gain-to-loss frequency in healthy controls, these effects were attenuated in individuals with schizophrenia (28). Furthermore, whereas higher IQ was associated with higher net scores towards the middle of the task (block 3 of 5) across individuals with schizophrenia and healthy controls, this effect was attenuated in individuals with schizophrenia earlier in the task (block 2) (28). A possible explanation for why IQ becomes more influential later during the IGT for individuals with schizophrenia may be that difficulties with executive function make them use more trials to decipher the contingencies of the task and correct their strategy accordingly. Whereas executive functions within the typical range may have a small or negligible impact on IGT performance (34), difficulties with executive functions may have a stronger impact on IGT performance (34, 41). First, difficulties with attending to the task (attention) make it difficult to code relevant information, and if coded, difficulties with holding the relevant information in mind to decipher the contingencies (working memory) may hinder the use of that information. Furthermore, if attending and holding the relevant information in mind, the participants still must inhibit the prepotent response to go for the decks with largest gains (response inhibition), shift focus from one deck to another (shifting) and plan a strategy as the relevant contingencies are deciphered (planning). Thus, difficulties with one or more executive functions may come into play when completing the IGT.

Overall, few studies (*k* = 6) of individuals with schizophrenia have examined the relationship between executive functions and performance on the IGT and related tasks (27). Moreover, the studies that have examined this relationship have often focused on complex tasks tapping a range of different executive functions at the same time, such as the Wisconsin Card Sorting Task, or just a specific executive function like working memory (27, 41). In order to gain a more refined understanding of how executive functions may contribute to IGT performance among individuals with schizophrenia, our aim was to examine the impact of a range of different executive functions including attention, response inhibition, shifting, working memory, and planning on IGT performance. We expected the different executive functions to have an impact on IGT performance beyond estimated IQ, and expected a composite score of all executive functions to have the most notable impact (16, 42). We also controlled for positive and negative symptoms, as previous studies have found a negative association between negative symptoms and decision-making (27). Before examining our main aim, we examined whether our participants with schizophrenia displayed the expected improvement from block 1 and onwards (i.e., learning trajectory), and their deck preferences.

## 2 Materials and methods

### 2.1 Procedures

Data for the current analysis was collected as part of a baseline assessment in a randomized controlled trial examining the effects of the metacognitive strategy training, Goal Management Training for executive functions, in a sample of persons with schizophrenia (43). The trial was preregistered at clinicaltrials.gov (NCT03048695) and approved by the Regional Committee for Medical and Health Research Ethics in Norway (2015/2118) prior to commencement. All participants gave informed consent in writing. The study took place at a regional hospital in Norway 2017-2021 and participants were recruited with the help of treating clinicians.

For this analysis, only the baseline data from participants with a schizophrenia spectrum disorder according to the criteria in the Diagnostic and Statistical Manual of Mental Disorders, DSM-IV-TR (44) were utilized. The diagnostic evaluation was performed by a clinical psychologist under supervision from a specialist in psychiatry using the Structured Clinical Interview for the Diagnostic and Statistical Manual of Mental Disorders-IV (DSM-IV) Axis 1 disorders, SCID I (45). A specialist in clinical neuropsychology supervised the cognitive assessment.

Inclusion criteria were age 16 - 69 years and subjective complaints of executive function difficulties. Exclusion criteria were (1) having received treatment for psychosis for longer than five years, (2) ongoing substance abuse, (3) neurological disease or traumatic brain injury, or (4) severe intellectual disability (IQ <70).

### 2.2 Participants

The participants in the sample (n = 65) comprised 40% females and 60% males who were aged 16 – 44 years (*M*_age_ = 25.4, *SD* = 6.5). The majority, 86.2%, were of European descent. See **Table 1** for a description of the sample.

**Table 1:** Sample characteristics.

*Description*	*Frequency*	*Mean*	*SD*	*SE*
Age		25.42	6.35	0.81
Sex				
Female	26 (40.0%)			
Male	39 (60.0%)			
Education in years		12.86	1.81	0.26
Diagnosis (DSM-IV):				
Schizophrenia	29 (44.6%)			
Schizoaffective disorder	14 (21.5%)			
Schizophreniform disorder	6 (9.2%)			
Psychotic disorder not otherwise specified	15 (23.1%)			
Delusional disorder	1 (1.5%)			
Duration of untreated psychosis (weeks)		241.18	244.01	30.27
Hospitalizations		3.23	5.07	0.63
Duration hospitalized in sum (months)		5.69	8.15	1.01
Drug therapy	51 (78.5%)			
Antipsychotics	45 (69.2%)			
Occupational status (n = 63)				
Ordinary full time work or study	12 (18.5%)			
Ordinary part time work or study	9 (13.8%)			
Supported employment	13 (20.0%)			
Disability benefits (n = 54)	13 (20.0%)			
Living situation				
Living independently (alone/flat share)	23 (35.4%)			
Living independently (with partner or children)	11 (16.9%)			
With parents (and siblings)	18 (27.7%)			
In supported housing	13 (20.0%)			

### 2.3 Measures

#### 2.3.1 Estimated IQ

The Matrix Reasoning and Vocabulary subtests from Wechsler Abbreviated Scale of Intelligence (WASI) or the General Ability Index from Wechsler’s Adult Intelligence Scale, 4th edition (WAIS-IV) were used as estimates of IQ (46, 47). Estimated IQ was used as a control variable in the regression analysis.

#### 2.3.2 Symptoms of psychosis

The severity of psychotic symptoms was assessed at the time of testing, using the Structured Clinical Interview for the Positive and Negative Syndrome Scale for Schizophrenia (SCI-PANSS), which includes a structured interview with participants, supplemental information from caregivers, and clinical observations made by mental health professionals (48). Symptom severity is measured on a scale ranging from 1 (*absent*) to 7 (*extreme*). A score of 4 is considered above the psychotic threshold for the items covering hallucinations and delusions. The inter-rater reliability of the Norwegian version of the instrument is adequate when it is performed by trained clinicians (49). In the present study the total score for seven positive symptom items and the total score for seven negative symptoms according to the original scale was utilized, as this allows for comparison with previous studies. Positive and negative symptoms were controlled for in the regression analysis.

#### 2.3.3 Response inhibition and shifting

The time raw scores on the Color-Word Interference Test (CWIT), from the Delis-Kaplan Executive Function System (D-KEFS (50), condition 3 (CW3; response inhibition) and condition 4 (CW4; shifting) were used as measures of response inhibition and shifting, respectively. In the CWIT, the participant is presented with color words with dissonant ink (e.g., the word “red” written with blue ink) and asked to name the dissonant color (condition 3) instead of reading the word, or to switch back-and-forth between reading the word and naming the dissonant color (condition 4). The CWIT condition 3 and 4 have shown adequate test-retest reliability (*r* = .52 to.90) in a general population sample (50, 51) as well as discriminative validity in differentiating between populations with and without difficulties with executive functions (15, 52). CW3 and CW4 were used as independent variables in the regression analyses independent variables in the regression analyses to examine the influence of response inhibition and shifting on IGT performance.

#### 2.3.4 Attention

The detectability score of the Conners Performance Test, 3^rd^ edition (CPT-3), was used as a measure of an individual’s sustained attention and discriminative processing, offering insight into their executive functions regarding focus and response consistency (53). In the CPT-3, participants were instructed to respond quickly to letters appearing on a screen by pressing a button for all letters appearing, while refraining from pressing the button when the letter X appeared. The test comprises 360 trials, whereof 20% of them present the letter X. The detectability score is calculated as the ratio of incorrect responses to the non-target (i.e., the letter X) divided by correctly identified targets. The CPT-3 scores have shown good test-retest reliability (*r* ≥.74) in a general population sample (53) as well as discriminative validity in differentiating between populations with and without attention deficits (54, 55). CPT detectability was used as an independent variable in the regression analyses to examine the IGT performance.

#### 2.3.5 Planning

The Tower test from the Delis-Kaplan Executive Function System (D-KEFS (50); was used as a measure of planning. In the Tower test, the participant is asked to move five disks in order to reproduce a target tower, varying in complexity, across three pegs. The participant is only allowed to move one disk at a time and is instructed to use as few moves as possible. We used the total achievement score, which is comprised by a combination of the time the participants use and the number of moves. The Tower test has shown weak test-retest reliability (*r* = .41 to.51) in a general population sample (50). However, the test has shown convergent validity through significant association with the Tower of London test (56) and discriminative validity in differentiating between individuals with and without traumatic brain injury (57). The Tower total achievement score was used as an independent variable in the regression analyses to examine the influence of planning on IGT performance.

#### 2.3.6 Working memory

The Letter-Number Sequencing (LNS) test from the Wechsler Intelligence Scale for Children (58) was used as a measure of working memory. In the LNS test, the participant is asked to recall a sequence of letters and numbers read aloud by the test administrator and thereafter repeated the numbers in ascending order and the letters in alphabetic order. The LNS has shown adequate test-retest reliability (*r* ≥.69) (59–61) as well as discriminative validity in differentiating between populations with and without difficulties with executive functions (62) and predictive validity in relation to occupational attainment in patients with psychosis (63). The LNS total score was used as an independent variable in the regression analyses of whether working memory contributed to IGT performance.

#### 2.3.7 Composite score of executive functioning

In addition to the test scores on the specific cognitive tasks, we also calculated a composite score of executive functioning for use in the analyses. The composite measure of executive functioning was added because there is an ongoing debate about whether executive functioning should be considered a unidimensional or multidimensional construct (64, 65). Furthermore, several studies have suggested that composite measures of executive functioning have greater clinical utility and higher predictive value and reliability (16, 66, 67). A composite measure also fits with the proposition that different specific cognitive processes may contribute to explaining individual differences in IGT performance (36). We created the composite score by converting all test scores to standardized scores (i.e., Z-scores) and then adding the Z-scores into a composite score of executive functioning. Higher scores on the composite reflect poorer executive functioning. The composite score of executive functioning was considered the primary independent variable in the regression analyses to examine the contribution of executive functioning to performance on the IGT.

#### 2.3.8 Decision-making

A computerized version of the IGT, the IGT version 2 (33, 68), was used as a measure of decision-making. The participants were instructed that the goal of the task was to maximize their gains through choosing cards form the different decks (A, B, C, and D). In the IGT, the participant is presented with four decks of cards with varying gains and losses and is asked to maximize gain by choosing between cards from the four decks across five blocks of 20 trials. Two of the decks (A and B) are associated with larger gains but even greater losses, leading to a net loss over time, whereas the two other decks (C and D) are associated with smaller gains but even smaller losses, leading to a net gain over time. Decks A and C are characterized by frequent and smaller losses, whereas deck B and D is characterized by less frequent but larger losses. The participant is not informed about these probabilities and must decipher the contingencies of the task themselves. The gain-to-loss structure of the task can be seen in **Table 2**. The participants were instructed that some decks may be more beneficial than others, but not which decks. The task ended after five blocks of 20 trials (a total of 100 trials). We collected information about the participants total net score, as well as net scores of the five blocks, and the number of responses to each deck. The IGT has shown adequate internal consistency across blocks (α = .75) in previous studies (32, 69) and is considered to have adequate construct validity in terms of differentiating between clinical and non-clinical populations (70). The test-retest reliability of the IGT has been questioned as one study found the IGT to display low test-retest reliability (*r* = .26-.27) over a three-week period (71). However, a recent study found similar rank-order stability (*r* = .25) over an eight-year period across a clinical and non-clinical population (32), suggesting at least some stability in performance over time.

**Table 2:** Gain-to-loss structure of Iowa Gambling Task, version 2. First twenty draws.

Draw number	Deck A	Deck B	Deck C	Deck D
1	+100	+100	+50	+50
2	+120	+80	+60	+40
3	+80, **-150**	+110	+40, **-50**	+45
4	+90	+120	+55	+45
5	+110, **-300**	+90	+55, **-50**	+55
6	+100	+100	+45	+60
7	+80, **-200**	+90	+50, **-50**	+40
8	+120	+120	+45	+55
9	+110 **-250**	+110, **-1250**	+60, **-50**	+50
10	+90, **-350**	+80	+40, **-50**	+60, **-250**
11	+110	+110	+55	+55
12	+130, **-350**	+100	+55, **-25**	+40
13	+90	+90	+65, **-75**	+60
14	+100, **-250**	+130, **-1500**	+45	+40
15	+120, **-200**	+120	+70, **-25**	+45
16	+130	+130	+40	+55
17	+90, **-300**	+110	+50, **-25**	+65
18	+130, **-150**	+90	+60, **-75**	+70
19	+120, **-250**	+100	+70	+50
20	+100	+120	+40, **-50**	+70, **-275**

Bold values mean losses.

Two measures of performance on the IGT were used in the analysis: The total net score and a measure of gain-to-loss frequency. The total net score, often referred to as expectancy value, is calculated by subtracting the number of draws from decks with lower expectancies of winning money in the long term due to larger losses (the so-called ‘bad’ decks A and B) from the number of draws from decks with higher long-term win expectancies (the so-called ‘good’ decks C and D). As a measure of preference for decks with high gain-to-loss frequency, the number of draws from decks A and C were subtracted from draws from decks B and D. Both the net score and the gain-to-loss frequency were used as the dependent variable in separate regression analyses of the contribution of executive functions on IGT performance, controlling for estimated IQ and symptoms of psychosis.

### 2.4 Data analyses

We performed all analyses in SPSS version 29. First, we used repeated-measures analysis of variance (ANOVA) to examine the learning trajectory across the five blocks of the IGT and deck preferences across the four decks of the IGT. Second, we examined bivariate correlations between the different cognitive processes, IGT performance, and psychosis symptoms using Pearson’s product-moment correlation coefficient (*r*). Third, we examined the contribution of each of the specific cognitive processes to IGT performance beyond estimated IQ and psychosis symptoms in hierarchical regression analyses. In step 1, we entered psychosis symptoms and estimated IQ as predictors to control for these effects in the subsequent step. In step 2, we entered the specific cognitive tasks, as well as a composite measure of executive functioning, in separate analyses. We used the increase in explained variance (Δ*R^2^*) to determine model fit and set the significance level (α) at *p* ≤.05. We assumed missing data were missing at random and dealt with missingness using listwise deletion. A *post hoc* power analysis showed that all stepwise regression analyses achieved an acceptable power of ≥.79 to detect a medium increase in explained variance (*f^2^* = .15, Δ*R^2^* = .13) from step 1 to step 2 (72, 73). An *f^2^* of respectively 0.02, 0.15, and 0.35 were considered a small, medium, and large effect size (74).

After finding a significant effect of estimated IQ on the IGT total net score, we did a *post hoc* visual inspection of the learning trajectory across IGT blocks according to IQ. We divided participants into three groups based on their normed IQ estimates; 1) a group with an average estimated IQ of the normative mean of 100 or above, 2) a group with estimated IQ between 99 and 1 SD below the normative mean (85), and 3) a group with an average estimated IQ between 1 and 2 SD below the normed mean (70–84).

## 3 Results

### 3.1 Learning trajectory and deck preferences

**Figure 1:**
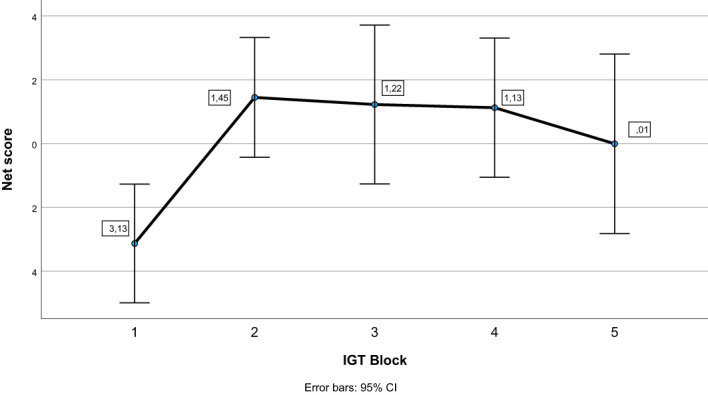
Net scores across blocks on the Iowa Gambling Task (IGT). Error bars display 95% confidence intervals.

**Figure 2:**
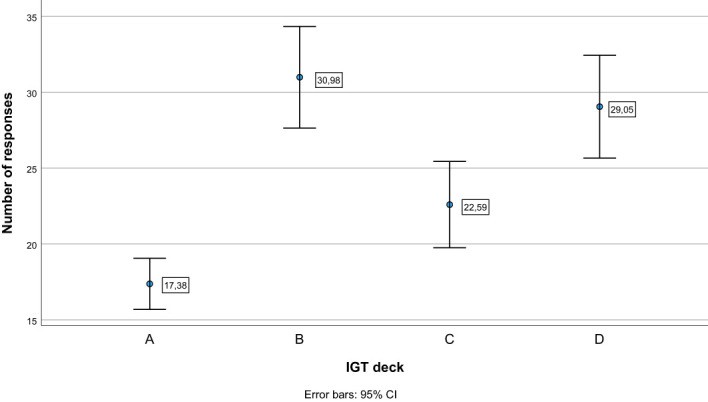
Number of responses to each deck on the Iowa Gambling Task (IGT). Error bars display 95% confidence intervals. (A) and (B) = Disadvantageous decks with high gains, but even higher losses. (C) and (D) = Advantageous decks with low gains, but even lower losses. Decks B and D have less frequent losses (18:2 per block), compared to decks (A) and (C) (10:10 per block).

See **Table 3** for descriptive statistics. The repeated-measures ANOVA with block as within-subject factor showed an overall significant effect of block (Pillai’s Trace = .233, *F (* 4, 60) = 4.565, *p* = .003). As can be seen in **Figure 1**, pairwise comparisons showed that the net score in block 1 differed significantly from the net score of all other blocks (*p ≤.*009), whereas there were no significant differences in net score between blocks 2, 3, 4, and 5 (*p* ≥.336). **Figure 2** illustrates mean number of responses to the IGT decks in the sample. The repeated-measures ANOVA with deck as a within-subject factor showed an overall significant effect of deck (Pillai’s Trace = .633, *F (* 4, 60) = 35.137, *p* <.001). Deck B received significantly more responses compared to deck A (*MD* = 13.61, *p* <.001) and deck C (*MD* = 8.39, *p* = .003). Deck D received significantly more responses compared to deck A (*MD* = 11.67, *p* <.001) and deck C (*MD* = 6.45, *p* = .010). Deck C received significantly more responses compared to deck A (*MD* = 5.22, *p* = .005). This suggests an emphasis on gain-to-loss frequency, as the two decks where participants win more often and lose more seldom (B and D) were chosen significantly more often than decks where losses occur more frequently (A and C).

**Table 3:** Descriptive statistics on the measures included in the current study.

	*Mean*	*SD*	Range	*n*
1. IGT net score (C+D)-(A+B)	3.47	31.67	-66.00, 72.00	64
2. IGT gain:loss frequency (B+D)-(A+C)	20.06	24.30	-40,00, 68,00	64
3. PANSS total positive symptoms	18.26	0.50	9.00, 29.00	65
4. PANSS total negative symptoms	17.77	0.60	7.00, 28.00	65
5. Estimated IQ	98.03	13.97	70, 131	60
6. CW3	63.63	22.96	39, 153	63
7. CW4	70.02	21.81	42, 144	63
8. CPT detect	-2.40	.90	-4.19, -.26	64
9. Tower	17.77	3.86	7, 30	65
10. LNS	18.21	3.76	5, 30	61
11. EF composite	-.08	3.05	-4.71, 8.75	58

PANSS, Positive and Negative Syndrome Scale. Estimated IQ, Wechsler Abbreviated Scale of Intelligence or the General Ability Index from Wechsler’s Adult Intelligence Scale, 4th edition. CW, Color-Word Interference Test; LNS, Letter-Number Sequencing Task; EF, Executive Function.

### 3.2 Cognitive processes and IGT performance

**Figure 3:**
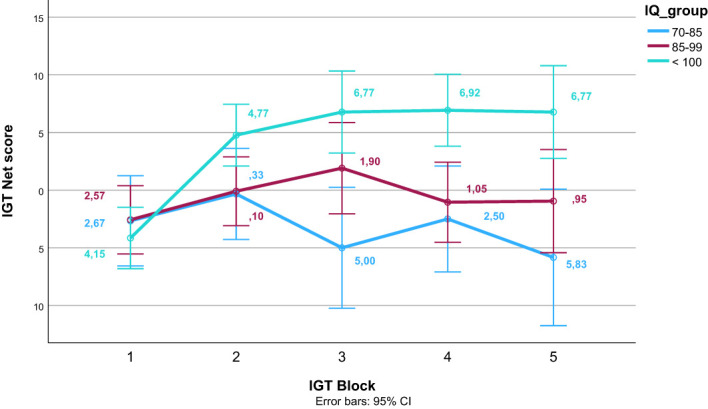
Net scores on the Iowa Gambling Task (IGT) across block for different levels of estimated intelligence quotient (IQ). Error bars display 95% confidence intervals.

**Table 4** presents the bivariate correlations between the different specific cognitive tasks, the composite score of executive functioning, estimated IQ, psychosis symptoms, and IGT performance (total net score and gain-to-loss frequency). IGT total net score correlated significantly with estimated IQ, response inhibition (CW3), and the composite score of executive functioning. IGT gain-to-loss frequency correlated significantly with the composite score of executive functioning. **Table 5** presents the results from the stepwise regression analyses with the IGT total net score as the dependent variable. After adding psychosis symptoms and estimated IQ in step 1, none of the cognitive tasks nor the composite score of executive functioning significantly contributed to increased explained variance in the IGT total net score in step 2. Estimated IQ was the only significant predictor of the IGT total net score, and consistently predicted this score across all analyses with differing executive tasks. In step 1, psychosis symptoms and estimated IQ together explained 28% of the variance in IGT performance (*F* (3, 55) = 8.492, *p* <.001, *R^2^* = .317, Adjusted *R^2^* = .279, *f* ^2^ = .46). As can be seen in the illustration in **Figure 3**, those with lower estimated IQ showed little or no progression in terms of increasing net scores across blocks compared to those with higher estimated IQ who clearly increased their net score from block 2 onwards. **Table 6** presents the results from the stepwise regression analyses with the IGT gain-to-loss frequency as the dependent variable. In this analysis, IQ and symptoms of psychosis did not explain variation in IGT gain-to-loss frequency, (*F* (3, 49) = .139, *p* = .936, *R^2^* = .008, Adjusted *R^2^* = -.052, *f* ^2^ = .01). However, adding the composite measure of executive functioning in step 2 significantly increased the explanatory power of the model with a small, but significant change in explained variance (Δ*F* (4, 48) = 5.256, *p*.026, *R^2^* = .106, Adjusted *R^2^* = .032, Δ*R^2^* = .098, *f* ^2^ = .12). The individual components of executive functioning did not reach statistical significance when entered as predictors of IGT gain-to-loss frequency.

**Table 4:** Correlation-matrix displaying the bivariate correlations between cognitive tasks, IQ, psychosis symptoms, and IGT performance.

	1.	2.	3.	4.	5.	6.	7.	8.	9.	10.
1. IGT net score	–									
2. IGT gain:loss	-.20	–	–							
3. PANSS pos.	-.06	-.07		–						
4. PANSS neg.	.02	-.07	.17	–	–					
5. Estimated IQ	.54***	.04	-.17	-.20	–	–				
6. CW3	-.27*	-.21	.10	.06	-.33*	–	–			
7. CW4	-.22	.22	.09	.04	-.23	.78***	–	–		
8. CPT detect	-.20	-.10	.05	.04	-.37**	.22	.27*	–	–	
9. Tower	-.03	-.21	-.07	-.15	-.07	.17	.12	.01	–	–
10. LNS	-.23	-.12	.09	.07	-.52***	.15	.13	.21	.07	–
11. EF comp.	-.35**	-.27*	.03	.06	-.48***	.76***	.76***	.58***	.43***	.52***

*p ≤.05, **p ≤.01, ***p ≤.001. IGT, Iowa Gambling Task; net score, decks (C+D)-(A+B); IGT gain-to-loss frequency, decks (B+D)-(A+C). PANSS pos., Positive and Negative Syndrome Scale; positive symptoms subscale. PANSS neg., Positive and Negative Syndrome Scale; negative symptoms subscale. Estimated IQ, Wechsler Abbreviated Scale of Intelligence or the General Ability Index from Wechsler’s Adult Intelligence Scale, 4th edition. CW, Color-Word Interference Test; LNS, Letter-Number Sequencing Task; EF comp., Executive Function Composite Score.

**Table 5:** Results from stepwise regression analyses with Iowa Gambling Task (IGT) performance measured by the total net score as dependent variable.

	Step 1			Step 2			
Predictors	*B*	*SE*	*p*	*B*	*SE*	*p*	Δ*R^2^*
EF composite							
PANSS pos.	.160	1.007	.875	.068	1.011	.946	
PANSS neg.	.930	.784	.241	.849	.789	.287	
Estimated IQ	1.428	.283	**<.001**	1.270	.325	**<.001**	
EF composite				-4.054	4.137	.332	.013
Response inhibition							
PANSS pos.	.585	.874	.507	.620	.878	.483	
PANSS neg.	.817	.771	.294	.813	.773	.298	
Estimated IQ	1.448	.269	**<.001**	1.373	.284	**<.001**	
CW3				-3.136	3.631	.392	.009
Shifting							
PANSS pos.	.585	.874	.507	.659	.872	.453	
PANSS neg.	.817	.771	.294	.801	.767	.301	
Estimated IQ	1.448	.269	**<.001**	1.379	.274	**<.001**	
CW4				-4.360	3.532	.223	.018
Attention/vigilance							
PANSS pos.	.435	.883	.624	.443	.891	.621	
PANSS neg.	.923	.780	.242	.896	.793	.263	
Estimated IQ	1.330	.264	**<.001**	1.300	.287	**<.001**	
CPT detection				-1.083	3.855	.780	.001
Planning							
PANSS pos.	.435	.883	.624	.472	.899	.602	
PANSS neg.	.923	.780	.242	.948	.792	.236	
Estimated IQ	1.330	.264	**<.001**	1.336	.267	**<.001**	
Tower				1.094	3.793	.774	.001
Working memory							
PANSS pos.	-.057	1.014	.955	-.057	1.024	.956	
PANSS neg.	1.019	.796	.206	1.030	.810	.209	
Estimated IQ	1.293	.275	**<.001**	1.313	.326	**<.001**	
LNS				.493	4.275	.909	.000

**Bold** text, significant p-values. *B*, unstandardized regression coefficient; *SE*, standard error of the regression coefficient; *p*; statistical significance; Δ*R^2^*, Change in explained variance with step 2 of the model. PANSS, Positive and Negative Syndrome Scale; Pos., Positive symptoms subscale; Neg., Negative symptoms subscale; IQ, Intelligence Quotient; EF, Executive Function Composite Score; CW, Color-Word Interference Test; CPT, Connors Continuous Performance Test; LNS, Letter-Number Sequencing Task.

**Table 6:** Results from stepwise regression analyses with Iowa Gambling Task (IGT) performance measured as selection of decks with higher gain-to-loss frequency as the dependent variable.

	Step 1			Step 2			
Predictors	*B*	*SE*	*p*	*B*	*SE*	*p*	Δ*R^2^*
EF composite							
PANSS pos.	-1.94	6.43	.764	-3.25	6.20	.602	
PANSS neg.	-2.54	5.01	.614	-3.71	4.83	.447	
Estimated IQ	-.03	.258	.912	-.35	.29	.222	
EF composite				-8.30	3.62	**.026**	**.098**
Response inhibition							
PANSS pos.	-4.30	5.61	.447	-3.85	5.52	.488	
PANSS neg.	-3.24	4.95	.515	-3.29	4.86	.501	
Estimated IQ	-.09	.25	.708	-.23	.26	.379	
CW3				-5.58	3.26	.093	.052
Shifting							
PANSS pos.	-4.30	5.61	.447	-3.86	5.61	.495	
PANSS neg.	-3.24	4.95	.515	-3.34	4.93	.502	
Estimated IQ	-.09	.25	.708	-.15	.25	.551	
CW4				-3.68	3.25	.262	.024
Attention/vigilance							
PANSS pos.	-3.07	5.83	.601	-2.94	5.87	.618	
PANSS neg.	-2.62	5.16	.614	-3.03	5.22	.564	
Estimated IQ	.02	.25	.953	-.05	.27	.847	
CPT detection				-2.38	3.63	.516	.008
Planning							
PANSS pos.	-3.07	5.83	.601	-4.60	5.77	.428	
PANSS neg.	-2.62	5.16	.614	-3.67	5.08	.473	
Estimated IQ	.02	.25	.953	-.02	.24	.932	
Tower				-6.42	3.48	.070	.059
Working memory							
PANSS pos.	-.14	6.66	.983	-.15	6.70	.982	
PANSS neg.	-1.84	5.23	.727	-2.22	5.30	.677	
Estimated IQ	.09	.26	.724	-.01	.31	.976	
LNS				-2.53	4.00	.530	.008

Bold text, significant p-values. *B*, unstandardized regression coefficient; *SE*, standard error of the regression coefficient; *p*, statistical significance; Δ*R^2^*, Change in explained variance with step 2 of the model. PANSS, Positive and Negative Syndrome Scale; Pos., Positive symptoms subscale; Neg., Negative symptoms subscale; IQ, Intelligence Quotient; EF, Executive Function Composite Score; CW, Color-Word Interference Test; CPT, Connors Continuous Performance Test; LNS, Letter-Number Sequencing Task.

## 4 Discussion

The current study aimed at examining the impact of executive functions on IGT performance in individuals with schizophrenia. Contrary to our expectations, the findings show that executive functions did not contribute to IGT performance beyond estimated IQ when using the preference for advantageous decks (C+D) as the measure of IGT performance. However, executive function, and not IQ, predicted patterns of preference for decks with differing frequency of wins and losses, as participants with poorer overall performance on executive function tasks where more likely to choose the decks with more frequent losses (A+C). Thus, the findings support the notion that decision-making and executive functions are relatively separate cognitive domains (34). Yet both general and specific cognitive abilities may contribute during decision making processes.

When it comes to learning trajectory and deck preferences, our findings diverge from previous findings on two issues. First, Betz and colleagues concluded in their meta-analysis that the healthy controls displayed a steep learning trajectory on the IGT, whereas individuals with schizophrenia did not improve during the task (28). On the contrary, our findings showed that individuals with schizophrenia displayed the typical learning trajectory on the IGT; they used the first block to decipher the contingencies and thereafter performed significantly better in subsequent blocks. The lack of a control group prevented us from comparing the learning trajectory of individuals with schizophrenia to the learning trajectory of healthy controls. Thus, a clear conclusion about the divergence in findings compared to the meta-analysis by Betz and colleagues is not possible. However, it may be that individuals with schizophrenia display improvement during the task, but that they improve significantly less than healthy controls. This interpretations is in line with findings from an earlier study of IGT in participants with schizophrenia, where the steepest learning curve was seen from block one to block two, but the participants with schizophrenia held a level of advantageous to disadvantageous deck somewhat below the performance of healthy participants in the last four blocks (75).

Second, Betz and colleagues (28) also concluded that whereas deck preferences among healthy controls were driven by gain-to-loss frequency, shown by the number of responses to decks B and D, deck preferences among individuals with schizophrenia were driven mainly by immediate gains, shown by the number of responses to decks A and B. Similarly, the meta-analysis by Woodrow and colleagues (27) found that individuals with psychosis placed a greater weight on gains over losses compared to healthy controls, which typically results in choosing the decks with greater gains, i.e., deck A and B. In contrast, our findings showed a within-group preference for deck B and D relative to A and C, a preference pattern consistent with the typical preferences of healthy controls (28, 35). This finding is in line with at least one other study of persons with schizophrenia using the IGT (75). In the present study, those participants with poorer executive function showed a greater preference for deck A and C. Again, the lack of a control group prevented us from investigating whether the magnitude of the preferences differs between individuals with schizophrenia and healthy controls. Nonetheless, it is interesting that the current sample, comprising young adults with schizophrenia spectrum disorders having subjective executive functioning difficulties, display a rather typical learning trajectory and deck preferences on the task as a group, whereas atypical preference for decks with more frequent losses were seen more frequently among participants with larger general cognitive challenges or executive functioning difficulties. Still, it should be noted that in terms of risk, deck B and D are the two decks that have the highest risk within the disadvantageous and advantageous decks, respectively (36). Thus, the results are consistent with the notion of individuals with schizophrenia being risk-taking in their decision-making. However, risk-taking decision-making on the IGT may be the norm also for healthy controls (41). Deck B and D share the same properties in terms of gain-to-loss frequency, and an increasing number of studies suggest that gain-to-loss frequency may drive decision-making on the IGT in both healthy and psychiatric populations (41, 76). If this is the case, also healthy participants may more often than previously assumed be using a “win-stay, lose-shift”-strategy, indicating that they are not able to guard against poor long-term outcomes (77).

Our research explored the influence of IQ on IGT performance and found that higher IQ scores predicted better performance, consistent with past studies suggesting that the IGT reflects cognitive rather than emotional processes (78). This finding underscores the necessity of considering IQ as a potential confounding variable when using the IGT across several populations, both healthy and clinical. Exploring the relationship between IQ and decision-making in healthy populations could provide further insights. Future studies should incorporate control comparisons to determine whether the relationship between IQ and decision-making abilities is unique to schizophrenia or more broadly applicable.

When it comes to the impact of cognitive abilities on IGT performance, our hypothesis that specific cognitive abilities like executive functions would be associated with IGT performance beyond general cognitive abilities (i.e., IQ) was partially supported. Estimated IQ was the only significant correlate of IGT total net score in multivariate analyses, whereas overall executive functioning was a significant correlate of IGT gain-to-loss frequency. Our findings align with previous studies demonstrating an association between IQ and IGT performance, and lend support to a smaller number of studies showing an associations between executive functioning and IGT performance in people with schizophrenia (27, 28). Still, a great proportion of the variance in IGT performance remains unaccounted for after investigating symptoms of psychosis, estimated IQ and a broad battery of executive functioning tests. This supports the notion that IGT measures some other rather specific cognitive ability that may (or may not) be of clinical relevance (34).

Of interest, poorer overall executive functions predicted more draws from deck A and C relative to deck B and D. This is interesting because the overall pattern in the sample was a preference for deck B and D, and because deck A and C have a lower gain-to-loss frequency and is thus associated with lower risk than deck B and D (36). Consequently, our findings suggest that individuals with SZ and poorer executive functions exhibit a more risk-aversive decision-making style compared to individuals with SZ and better executive functions, after controlling for IQ. As such, it may be that risk-taking behaviors in individuals with SZ are more deliberate than previously thought and may not be a consequence of difficulties with regulating one’s own behavior and emotions (75). Interestingly, it has been argued that individuals on the autism spectrum is more deliberate in their decision-making style and thus, more risk-aversive (79, 80). As individuals with SZ and individuals on the autism spectrum display similar performance on executive functions, and an impairment compared to healthy controls (15), this may suggest that poorer executive functions (at least sometimes) contribute to a more risk-aversive decision-making style. Thus, whereas IQ may show a similar association with IGT performance across healthy and clinical populations (78), executive functions may be differentially associated with IGT performance across healthy and clinical populations (40). However, this hypothesis needs to be examined in future studies including a healthy comparison group.

The varied findings related to executive functions (i.e., net score versus gain-loss frequency) can be interpreted in light of the clinical cases of damage to the ventromedial prefrontal cortex (vmPFC) that were central to the development of the IGT (33). In these cases, Bechara and colleagues describe that the patients usually have intact intellectual (i.e., IQ) and problem-solving (i.e., executive functions) abilities, but exhibit a rather severe impairment in real-life decision-making – and on the IGT (33). The extent to which specific cognitive abilities play a role in determining IGT performance may depend on the pathophysiology of cortical networks involved in particular disorders. For example, one study found that executive functions were related to IGT performance among healthy participants and participants with lesions to the dorsolateral prefrontal cortex (dlPFC) but not among participants with lesions to the vmPFC (81). Reduced motivation and pleasure (anhedonia) are common negative symptoms of schizophrenia and found to be related to dampened decision value signals in the vmPFC (82). However, pathology of the dlPFC has been assumed to be central to the cognitive difficulties of individuals with schizophrenia (83). Thus, the differential role that executive functions play in overall IGT performance versus gain-to-loss frequency may indicate that different cortical regions are involved in the choices between advantageous versus disadvantageous decks and risky versus less-risky decks. It is important to note that whereas poorer executive functions may contribute to a risk-aversive decision-making style (i.e., more frequent draws from deck A and C), this decision-making style do not entail poorer overall decision-making (i.e., net score). In sum, current evidence implies that several cortical networks are involved in the pathophysiology of schizophrenia, complicating the interpretation of IGT results in this population (39, 83, 84). Thus, future research must continue to develop task paradigms aimed at teasing out specific cognitive processes. Importantly, to increase clinical relevance it is also essential to combine measures at the physiological, neuropsychological, and behavioral levels in the same studies.

Although IGT performance is assumed to be a proxy of real-life decision-making, the evidence of IGT being associated with real-life outcomes is limited (32, 70). Some evidence has suggested that IGT performance is related to social functioning in individuals with schizophrenia and risk-taking in everyday life in young adults with ADHD (27, 32). However, a recent study of decision-making tasks similar to the IGT found low ecological validity of the tasks across two general population samples (85). The decision-making tasks were not related to risk-taking in everyday life, operationalized as preventive health behaviors (e.g., wearing a mask) during the COVID-19 pandemic (85). Thus, more research is needed to establish clear links between IGT performance and clinical outcomes (e.g., psychotic relapse, remission, occupational functioning etc.) among individuals with schizophrenia.

### 4.1 Strengths and limitations

The strengths of the current study include a well-characterized clinical sample who had undergone testing with a comprehensive neuropsychological test battery. A major limitation of the current study is the lack of a control group preventing us from comparing the IGT performance of individuals with schizophrenia to that of healthy controls matched on IQ or another clinical group. Furthermore, the requirement that participants had to report subjective executive functioning difficulties to participate in the clinical trial means that our participants may have larger cognitive deficits on average compared to the general population of individuals with schizophrenia. At the same time, based on established norms, few participants scored in the clinical range on the neuropsychological tests (43, 86). Thus, the sample may not be representative of the whole population of individuals with schizophrenia spectrum disorders. Furthermore, due to the limited samples size, we did not control for sex differences in the regression analysis. However, a recent meta-analysis found that males tend to perform better than females on the IGT (87). In future studies, larger samples or pooling of data would help clarify whether sex differences are relevant to the relationships between general and specific cognitive functioning and decision-making. In our study, 69.2% of participants received antipsychotic treatment. According to the systematic review and meta-analysis by Woodrow et al. (27), low-dose antipsychotic treatment showed no impairments in decision-making, while medium to high doses and one antipsychotic-free study demonstrated moderate impairments, suggesting a possible curvilinear relationship. Consequently, the type and dosage of antipsychotic medication in our study may have influenced IGT performance. While the overall impact of second generation antipsychotic medication on cognitive functions is thought to be minor, the exact relationships are largely unknown (88). A last limitation is the cross-sectional nature of the study. Since IGT testing only took place at baseline, we could not examine how treatment may have affected IGT performance, or how IGT performance may affect the clinical course of individuals with schizophrenia. Furthermore, we lacked a measure of risk-taking in everyday life preventing us from establishing ecological validity.

### 4.2 Conclusion and clinical implications

The current study showed that individuals with schizophrenia improve across blocks in the IGT and display the typical preference for decks with highly frequent gains, and low frequent losses. Estimated IQ was strongly related to IGT total net score (choosing decks that are advantageous in the long run), whereas specific cognitive abilities contributed to the explained variance in choosing decks with more frequent gains than losses similar to how healthy participants often approach the task. In the real world, individuals with schizophrenia who possess higher cognitive functioning (i.e., estimated IQ and/or executive functioning) may be better equipped to weigh risks and benefits, anticipate the consequences of their actions, and engage in more adaptive decision-making processes. Moreover, knowledge of how these individuals approach risk and make decisions can assist caregivers and health professionals in creating environments that minimize potential harm while encouraging autonomy and independence. Clinical implications include the importance of taking both general cognitive difficulties, executive difficulties and decision-making difficulties into account when providing health care to individuals with schizophrenia, for instance by adapting information to aid informed treatment decisions. Standardized clinical assessment ought to, at the very least, include measures of general abilities and executive functioning, but preferably also measures that can shed light on real-life decision-making.

## Data Availability

The raw data supporting the conclusions of this article will be made available by the authors, without undue reservation.

## References

[1] American Psychiatric Association. Diagnostic and statistical manual of mental disorders (DSM-5). Washington, D.C: American Psychiatric Publishing (2013).

[2] McCutcheon RA Reis Marques T Howes OD. Schizophrenia—An overview. JAMA Psychiatry. (2020) 77:201–10. doi: 10.1001/jamapsychiatry.2019.3360 31664453

[3] Saha S Chant D Welham J McGrath J. A systematic review of the prevalence of schizophrenia. PloS Med. (2005) 2:e141. doi: 10.1371/journal.pmed.0020141 15916472 PMC1140952

[4] Subramaniam M Abdin E Vaingankar JA Sambasivam R Zhang YJ Shafie S. Lifetime prevalence and correlates of schizophrenia and other psychotic disorders in Singapore. Front Psychiatry. (2021) 12:650674/full. doi: 10.3389/fpsyt.2021.650674/full 33776823 PMC7991584

[5] Charlson FJ Ferrari AJ Santomauro DF Diminic S Stockings E Scott JG. Global epidemiology and burden of schizophrenia: findings from the global burden of disease study 2016. Schizophr Bull. (2018) 44:1195–203. doi: 10.1093/schbul/sby058 PMC6192504 29762765

[6] Chong HY Teoh SL Wu DBC Kotirum S Chiou CF Chaiyakunapruk N. Global economic burden of schizophrenia: a systematic review. Neuropsychiatr Dis Treat. (2016) 12:357–73. doi: 10.2147/NDT.S96649 PMC4762470 26937191

[7] Peralta V García de Jalón E Moreno-Izco L Peralta D Janda L Sánchez-Torres AM. Long-term outcomes of first-admission psychosis: A naturalistic 21-year follow-up study of symptomatic, functional and personal recovery and their baseline predictors. Schizophr Bull. (2022) 48:631–42. doi: 10.1093/schbul/sbab145 PMC9077430 34999894

[8] Castelein S Timmerman ME Investigators P van der Gaag M Visser E. Clinical, societal and personal recovery in schizophrenia spectrum disorders across time: states and annual transitions. Br J Psychiatry. (2021) 219:401–8. doi: 10.1192/bjp.2021.48 PMC8529640 35048855

[9] Javitt DC. Current and emergent treatments for symptoms and neurocognitive impairment in schizophrenia. Curr Treat Opt Psychiatry. (2014) 1:107–20. doi: 10.1007/s40501-014-0010-9 PMC4540407 26301175

[10] Green MF Horan WP Lee J. Nonsocial and social cognition in schizophrenia: current evidence and future directions. World Psychiatry. (2019) 18:146–61. doi: 10.1002/wps.20624 PMC6502429 31059632

[11] Fioravanti M Bianchi V Cinti ME. Cognitive deficits in schizophrenia: an updated metanalysis of the scientific evidence. BMC Psychiatry. (2012) 12:64. doi: 10.1186/1471-244X-12-64 22715980 PMC3528440

[12] Kendler KS Ohlsson H Sundquist J Sundquist K. IQ and schizophrenia in a Swedish national sample: their causal relationship and the interaction of IQ with genetic risk. Am J Psychiatry. (2015) 172:259–65. doi: 10.1176/appi.ajp.2014.14040516 PMC4391822 25727538

[13] Khandaker GM Barnett JH White IR Jones PB. A quantitative meta-analysis of population-based studies of premorbid intelligence and schizophrenia. Schizophr Res. (2011) 132:220–7. doi: 10.1016/j.schres.2011.06.017 PMC3485562 21764562

[14] Carruthers SP Van Rheenen TE Gurvich C Sumner PJ Rossell SL. Characterising the structure of cognitive heterogeneity in schizophrenia spectrum disorders. A systematic review and narrative synthesis. Neurosci Biobehav Rev. (2019) 107:252–78. doi: 10.1016/j.neubiorev.2019.09.006 31505202

[15] Øie MG Andersen PN Hovik KT Skogli EW Rund BR. Similar impairments shown on a neuropsychological test battery in adolescents with high-functioning autism and early onset schizophrenia: a two-year follow-up study. Cognit Neuropsy. (2020) 25:163–78. doi: 10.1080/13546805.2020.1713736 31931670

[16] Øie MG Sundet K Haug E Zeiner P Klungsøyr O Rund BR. Cognitive performance in early-onset schizophrenia and attention-deficit/hyperactivity disorder: A 25-year follow-up study. Front Psychol. (2021) 11. doi: 10.3389/fpsyg.2020.606365/full PMC7841368 33519613

[17] Orellana G Slachevsky A. Executive functioning in schizophrenia. Front Psychiatry. (2013) 4:35/full. doi: 10.3389/fpsyt.2013.00035/full 23805107 PMC3690455

[18] Sheffield JM Karcher NR Barch DM. Cognitive deficits in psychotic disorders: A lifespan perspective. Neuropsychol Rev. (2018) 28:509–33. doi: 10.1007/s11065-018-9388-2 PMC6475621 30343458

[19] Wobrock T Ecker UKH Scherk H Schneider-Axmann T Falkai P Gruber O. Cognitive impairment of executive function as a core symptom of schizophrenia. World J Biol Psychiatry. (2009) 10:442–51. doi: 10.1080/15622970701849986 18609418

[20] Carruthers SP Gurvich CT Meyer D Bank ASR Bousman C Everall IP. Exploring heterogeneity on the Wisconsin card sorting test in schizophrenia spectrum disorders: A cluster analytical investigation. J Int Neuropsychol Soc. (2019) 25:750–60. doi: 10.1017/S1355617719000420 31104647

[21] Diamond A. Executive functions. Annu Rev Psychol. (2013) 64:135–68. doi: 10.1146/annurev-psych-113011-143750 PMC4084861 23020641

[22] Dibben CRM Rice C Laws K McKenna PJ. Is executive impairment associated with schizophrenic syndromes? A meta-analysis. Psychol Med. (2009) 39:381–92. doi: 10.1017/S0033291708003887 18588741

[23] Schaefer J Giangrande E Weinberger DR Dickinson D. The global cognitive impairment in schizophrenia: Consistent over decades and around the world. Schizophr Res. (2013) 150:42–50. doi: 10.1016/j.schres.2013.07.009 23911259 PMC4196267

[24] de Gracia Dominguez M Viechtbauer W Simons CJP van Os J Krabbendam L. Are psychotic psychopathology and neurocognition orthogonal? A systematic review of their associations. Psychol Bull. (2009) 135:157–71. doi: 10.1037/a0014415 19210058

[25] Strugstad B Lau B Glenne Øie M. Associations between cognition and internalizing problems in young adults with early-onset schizophrenia: A 13-year follow-up study. Psychiatry Res. (2018) 265:161–6. doi: 10.1016/j.psychres.2018.04.033 29709790

[26] Øie M Sundet K Ueland T. Neurocognition and functional outcome in early-onset schizophrenia and attention-deficit/hyperactivity disorder: A 13-year follow-up. Neuropsychology. (2011) 25:25–35. doi: 10.1037/a0020855 21090901

[27] Woodrow A Sparks S Bobrovskaia V Paterson C Murphy P Hutton P. Decision-making ability in psychosis: a systematic review and meta-analysis of the magnitude, specificity and correlates of impaired performance on the Iowa and Cambridge Gambling Tasks. Psychol Med. (2019) 49:32–48. doi: 10.1017/S0033291718002660 30246669

[28] Betz LT Brambilla P Ilankovic A Premkumar P Kim MS Raffard S. Deciphering reward-based decision-making in schizophrenia: A meta-analysis and behavioral modeling of the Iowa Gambling Task. Schizophr Res. (2019) 204:7–15. doi: 10.1016/j.schres.2018.09.009 30262254

[29] Yee N Matheson S Korobanova D Large M Nielssen O Carr V. A meta-analysis of the relationship between psychosis and any type of criminal offending, in both men and women. Schizophr Res. (2020) 220:16–24. doi: 10.1016/j.schres.2020.04.009 32359974

[30] Khokhar JY Dwiel LL Henricks AM Doucette WT Green AI. The link between schizophrenia and substance use disorder: A unifying hypothesis. Schizophr Res. (2018) 194:78–85. doi: 10.1016/j.schres.2017.04.016 28416205 PMC6094954

[31] Fischer BA McMahon RP Kelly DL Wehring HJ Meyer WA Feldman S. Risk-taking in schizophrenia and controls with and without cannabis dependence. Schizophr Res. (2015) 161:471–7. doi: 10.1016/j.schres.2014.11.009 PMC4308438 25467541

[32] Orm S Pollak Y Fossum IN Andersen PN Øie MG Skogli EW. Decision-making and risky behavior in individuals with attention-deficit/hyperactivity disorder: A 10-year longitudinal study. Dev Neuropsychol. (2022) 47:193–209. doi: 10.1080/87565641.2022.2082430 35642565

[33] Bechara A Damasio AR Damasio H Anderson SW. Insensitivity to future consequences following damage to human prefrontal cortex. *Cognition* . (1994) 50(1):7–15. 10.1016/0010-0277(94)90018-3 8039375

[34] Toplak ME Sorge GB Benoit A West RF Stanovich KE. Decision-making and cognitive abilities: A review of associations between Iowa Gambling Task performance, executive functions, and intelligence. Clin Psychol Rev. (2010) 30:562–81. doi: 10.1016/j.cpr.2010.04.002 20457481

[35] Steingroever H Wetzels R Horstmann A Neumann J Wagenmakers EJ. Performance of healthy participants on the Iowa Gambling Task. Psychol Assess. (2013) 25:180–93. doi: 10.1037/a0029929 22984804

[36] Dekkers TJ Agelink van Rentergem JA Huizenga HM Raber H Shoham R Popma A. Decision-making deficits in ADHD are not related to risk seeking but to suboptimal decision-making: meta-analytical and novel experimental evidence. J Atten Disord. (2021) 25:486–501. doi: 10.1177/1087054718815572 30520666 PMC7783692

[37] Groen Y Gaastra GF Lewis-Evans B Tucha O. Risky behavior in gambling tasks in individuals with ADHD – A systematic literature review. PloS One. (2013) 8:e74909. doi: 10.1371/journal.pone.0074909 24058638 PMC3772864

[38] Damasio A. Descartes’ error: Emotion, rationality and the human brain. New York: Grosset/Putnam (1994).

[39] Bechara A Damasio H Tranel D Damasio AR. The Iowa Gambling Task and the somatic marker hypothesis: some questions and answers. Trends Cognit Sci. (2005) 9:159–62. doi: 10.1016/j.tics.2005.02.002 15808493

[40] Gansler DA Jerram MW Vannorsdall TD Schretlen DJ. Does the Iowa gambling task measure executive function? Arch Clin Neuropsychol. (2011) 26:706–17. doi: 10.1093/arclin/acr082 PMC3254153 22015855

[41] Xu M Lee WK Ko CH Chiu YC Lin CH. The prominent deck B phenomenon in schizophrenia: an empirical study on Iowa gambling task. Front Psychol. (2021) 12:619855. doi: 10.3389/fpsyg.2021.619855 34539474 PMC8446202

[42] Rund BR Barder HE Evensen J Haahr U Hegelstad W ten V Joa I. Neurocognition and duration of psychosis: A 10-year follow-up of first-episode patients. Schizophr Bull. (2016) 42:87–95. doi: 10.1093/schbul/sbv083 26101305 PMC4681546

[43] Haugen I Stubberud J Haug E McGurk SR Hovik KT Ueland T. A randomized controlled trial of Goal Management Training for executive functioning in schizophrenia spectrum disorders or psychosis risk syndromes. BMC Psychiatry. (2022) 22:575. doi: 10.1186/s12888-022-04197-3 36031616 PMC9420179

[44] American Psychiatric Association. Diagnostic and statistical manual of mental disorders: DSM-IV-TR. 4th ed. Washington, D.C: American Psychiatric Publishing (2000).

[45] First MB Spitzer RL Gibbon M Williams JB. Structured clinical interview for DSM-IV-TR Axis I disorders: patient edition. New York, NY, US: Biometrics Research Department, Columbia University (2005).

[46] Wechsler D. Wechsler Abbreviated Scale of Intelligence. New York: The Psychological Corporation (1999).

[47] Wechsler D. Wechsler Adult Intelligence Scale - Fourth Edition (WAIS-IV). San Antonio, Texas, US: Pearson Assessments (2008).

[48] Kay SR Fiszbein A Opler LA. The positive and negative syndrome scale (PANSS) for schizophrenia. Schizophr Bull. (1987) 13:261–76. doi: 10.1093/schbul/13.2.261 3616518

[49] Friis S Larsen TK Melle I Opjordsmoen S Johannessen JO Haahr U. Methodological pitfalls in early detection studies – the NAPE Lecture 2002. Acta Psychiatr Scand. (2003) 107:3–9. doi: 10.1034/j.1600-0447.2003.02600.x 12558535

[50] Delis D Kaplan E Kramer J. Delis-Kaplan Executive Function System (D-KEFS). Norwegian version. Stockholm: Pearson Assessments (2001).

[51] Homack S Lee D Riccio CA. Test review: Delis-kaplan executive function system. J Clin Exp Neuropsychol. (2005) 27:599–609. doi: 10.1080/13803390490918444 16019636

[52] Skogli EW Teicher MH Andersen PN Hovik KT Øie M. ADHD in girls and boys – gender differences in co-existing symptoms and executive function measures. BMC Psychiatry. (2013) 13:298. doi: 10.1186/1471-244X-13-298 24206839 PMC3827008

[53] Conners KC. Conners Continuous Performance Test. 3rd ed. Toronto: Multi-Health Systems Inc (2014).

[54] Ord AS Miskey HM Lad S Richter B Nagy K Shura RD. Examining embedded validity indicators in Conners continuous performance test-3 (CPT-3). Clin Neuropsychol. (2021) 35:1426–41. doi: 10.1080/13854046.2020.1751301 32364040

[55] Scimeca LM Holbrook L Rhoads T Cerny BM Jennette KJ Resch ZJ. Examining Conners continuous performance test-3 (CPT-3) embedded performance validity indicators in an adult clinical sample referred for ADHD evaluation. Dev Neuropsychol. (2021) 46:347–59. doi: 10.1080/87565641.2021.1951270 34256665

[56] Larochette AC Benn K Harrison AG. Executive functioning: A comparison of the tower of London DX and the D-KEFS tower test. Appl Neuropsychol. (2009) 16:275–80. doi: 10.1080/09084280903098695 20183182

[57] Lengenfelder J Arjunan A Chiaravalloti N Smith A DeLuca J. Assessing frontal behavioral syndromes and cognitive functions in traumatic brain injury. Appl Neuropsychol Adult. (2015) 22:7–15. doi: 10.1080/23279095.2013.816703 25529586

[58] Wechsler D. Wechsler Intelligence Scale for Children - Fourth edition: Norwegian version. Stockholm: The Psychological Corporation (2004).

[59] Baron IS. Test review: wechsler intelligence scale for children-fourth edition (WISC-IV). Child Neuropsychol. (2005) 11:471–5. doi: 10.1080/09297040590951587 16306021

[60] Lo AHY Humphreys M Byrne GJ Pachana NA. Test–retest reliability and practice effects of the Wechsler Memory Scale-III. J Neuropsychol. (2012) 6:212–31. doi: 10.1111/j.1748-6653.2011.02023.x 22257421

[61] San Miguel Montes LE Allen DN Puente AE Neblina C. Validity of the WISC–IV Spanish for a clinically referred sample of Hispanic children. Psychol Assess. (2010) 22:465–9. doi: 10.1037/a0018895 20528073

[62] Andersen PN Skogli EW Hovik KT Geurts H Egeland J Øie M. Working memory arrest in children with high-functioning autism compared to children with attention-deficit/hyperactivity disorder: Results from a 2-year longitudinal study. Autism. (2015) 19:443–50. doi: 10.1177/1362361314524844 24604922

[63] Murtagh A Hurley AL Kinsella A Corvin A Donohoe G Gill M. The Letter-Number Sequencing Test and its association with potential to work among people with psychotic illness. Eur Psychiatry. (2010) 25:101–4. doi: 10.1016/j.eurpsy.2009.06.004 19720503

[64] Karr JE Areshenkoff CN Rast P Hofer SM Iverson GL Garcia-Barrera MA. The unity and diversity of executive functions: A systematic review and re-analysis of latent variable studies. Psychol Bull. (2018) 144:1147–85. doi: 10.1037/bul0000160 PMC6197939 30080055

[65] Miyake A Friedman NP. The nature and organization of individual differences in executive functions: four general conclusions. Curr Dir Psychol Sci. (2012) 21:8–14. doi: 10.1177/0963721411429458 22773897 PMC3388901

[66] Orm S Andersen PN Teicher MH Fossum IN Øie MG Skogli EW. Childhood executive functions and ADHD symptoms predict psychopathology symptoms in emerging adults with and without ADHD: a 10-year longitudinal study. Res Child Adolesc Psychopathol. (2023) 51:261–71. doi: 10.1007/s10802-022-00957-7 PMC9867664 36194356

[67] Suchy Y Brothers SL. Reliability and validity of composite scores from the timed subtests of the D-KEFS battery. Psychol Assess. (2022) 34:483–95. doi: 10.1037/pas0001081 35298217

[68] Bechara A. Iowa Gambling Task™, Version 2. Lutz: PAR (2016).

[69] Skogli EW Andersen PN Hovik KT Øie M. Development of hot and cold executive function in boys and girls with ADHD: A 2-year longitudinal study. J Atten Disord. (2017) 21:305–15. doi: 10.1177/1087054714524984 24626329

[70] Buelow MT Suhr JA. Construct validity of the Iowa gambling task. Neuropsychol Rev. (2009) 19:102–14. doi: 10.1007/s11065-009-9083-4 19194801

[71] Buelow MT Barnhart WR. Test–retest reliability of common behavioral decision making tasks. Arch Clin Neuropsychol. (2018) 33:125–9. doi: 10.1093/arclin/acx038 28430836

[72] Faul F Erdfelder E Lang AG Buchner A. G*Power 3: A flexible statistical power analysis program for the social, behavioral, and biomedical sciences. Behav Res Methods. (2007) 39:175–91. doi: 10.3758/BF03193146 17695343

[73] Faul F Erdfelder E Buchner A Lang AG. Statistical power analyses using G*Power 3.1: Tests for correlation and regression analyses. Behav Res Methods. (2009) 41:1149–60. doi: 10.3758/BRM.41.4.1149 19897823

[74] Cohen J. A power primer. Psychol Bull. (1992) 112:155–9. doi: 10.1037/0033-2909.112.1.155 19565683

[75] Pedersen A Göder R Tomczyk S Ohrmann P. Risky decision-making under risk in schizophrenia: A deliberate choice? J Behav Ther Exp Psychiatry. (2017) 56:57–64. doi: 10.1016/j.jbtep.2016.08.004 27568887

[76] Brown EC Hack SM Gold JM Carpenter WT Fischer BA Prentice KP. Integrating frequency and magnitude information in decision-making in schizophrenia: An account of patient performance on the Iowa Gambling Task. J Psychiatr Res. (2015) 66–67:16–23. doi: 10.1016/j.jpsychires.2015.04.007 PMC4458199 25959618

[77] Lin CH Chiu YC Lee PL Hsieh JC. Is deck B a disadvantageous deck in the Iowa Gambling Task? Behav Brain Funct. (2007) 3:16. doi: 10.1186/1744-9081-3-16 17362508 PMC1839101

[78] Demaree HA Burns KJ DeDonno MA. Intelligence, but not emotional intelligence, predicts Iowa Gambling Task performance. Intelligence. (2010) 38:249–54. doi: 10.1016/j.intell.2009.12.004

[79] South M Chamberlain PD Wigham S Newton T Le Couteur A McConachie H. Enhanced decision making and risk avoidance in high-functioning autism spectrum disorder. Neuropsychology. (2014) 28:222–8. doi: 10.1037/neu0000016 24219603

[80] Brosnan M Lewton M Ashwin C. Reasoning on the autism spectrum: A dual process theory account. J Autism Dev Disord. (2016) 46:2115–25. doi: 10.1007/s10803-016-2742-4 PMC4860198 26960339

[81] Ouerchefani R Ouerchefani N Allain P Ben Rejeb MR Le Gall D. Relationships between executive function, working memory, and decision-making on the Iowa Gambling Task: Evidence from ventromedial patients, dorsolateral patients, and normal subjects. J Neuropsychol. (2019) 13:432–61. doi: 10.1111/jnp.12156 29667317

[82] Souther MK Wolf DH Kazinka R Lee S Ruparel K Elliott MA. Decision value signals in the ventromedial prefrontal cortex and motivational and hedonic symptoms across mood and psychotic disorders. NeuroImage Clin. (2022) 36:103227. doi: 10.1016/j.nicl.2022.103227 36242852 PMC9668619

[83] Smucny J Dienel SJ Lewis DA Carter CS. Mechanisms underlying dorsolateral prefrontal cortex contributions to cognitive dysfunction in schizophrenia. Neuropsychopharmacology. (2022) 47:292–308. doi: 10.1038/s41386-021-01089-0 34285373 PMC8617156

[84] Luvsannyam E Jain MS Pormento MKL Siddiqui H Balagtas ARA Emuze BO. Neurobiology of schizophrenia: A comprehensive review. Cureus. (2022). https://www.cureus.com/articles/92077-neurobiology-of-schizophrenia-a-comprehensive-review. 10.7759/cureus.23959 PMC9080788 35541299

[85] Buelow MT Okdie BM Kowalsky JM. Ecological validity of common behavioral decision making tasks: evidence across two samples. J Clin Exp Neuropsychol. (2024) 0:1–20. doi: 10.1080/13803395.2024.2337759 38591953

[86] Haugen I Stubberud J Ueland T Haug E Øie MG. Executive dysfunction in schizophrenia: Predictors of the discrepancy between subjective and objective measures. Schizophr Res Cogn. (2021) 26:100201. doi: 10.1016/j.scog.2021.100201 34189060 PMC8217703

[87] Zanini L Picano C Spitoni GF. The Iowa gambling task: men and women perform differently. A meta-analysis. Neuropsychol Rev. (2024). doi: 10.1007/s11065-024-09637-3 PMC11965174 38462590

[88] Allott K Chopra S Rogers J Dauvermann MR Clark SR. Advancing understanding of the mechanisms of antipsychotic-associated cognitive impairment to minimise harm: a call to action. Mol Psychiatry. (2024) 29:2571–4. doi: 10.1038/s41380-024-02503-x PMC11412898 38454078

